# Introduction to Special Issue on “Electromagnetic Technologies for Medical Diagnostics: Fundamental Issues, Clinical Applications and Perspectives”

**DOI:** 10.3390/diagnostics9010019

**Published:** 2019-02-13

**Authors:** Panagiotis Kosmas, Lorenzo Crocco

**Affiliations:** 1 Faculty of Natural and Mathematical Sciences, King’s College London, Strand, London WC2R 2LS, UK; 2 Institute for Electromagnetic Sensing of the Environment, National Research Council of Italy (IREA-CNR), 80124 Napoli, Italy

## 1. Motivation for the Special Issue

The application of microwave technologies in medical imaging and diagnostics is an emerging topic within the electromagnetic (EM) engineering community. Technological developments in this area have been accelerated by advances in antenna design and fabrication, computational methods, imaging theory and algorithms, as well as measurement techniques. Parallel to these developments, advancements in telecommunication industries have increased the capabilities and driven down the cost and form factor of microwave equipment. These important developments are paving the way for a new generation of low-cost, portable, and accurate microwave sensing/imaging systems, which could tackle various current challenges in medical diagnostics.

Microwave medical imaging exploits the possibility of a significant dielectric contrast between healthy and disease-affected tissues to detect a pathological condition. Arguably, breast cancer detection has been the most popular microwave medical imaging application in the last twenty years [1]; many techniques and systems have now advanced to clinical prototypes while also focusing on reducing cost using custom-made electronics [2]. Several research groups have also been investigating the possibility of using microwave imaging for various aspects of stroke treatment [3], including start-up companies built around this idea [4]. A number of review articles, such as [5], have presented the challenges and opportunities of these and other microwave imaging (as well as sensing) applications. In microwave sensing, the objective is not to produce diagnostic images of the body but, rather, to use microwave technology to monitor physiological parameters such as heart-rate [6], or disease-related biomarkers, such as glucose, in the blood [7]. These are just a few examples of what is becoming a very broad area of research, and we hope that this special issue can introduce this area to a new audience engaged in more traditional medical diagnostics.

## 2. Overview of Contributions

These eleven manuscripts cover many different topics and applications, ranging from reviews of the current state of the art to tools for their development, or techniques to tackle specific issues. In [8], for example, Joachimowicz et al. present novel experimental breast and head phantoms, fabricated from 3D-printed structures, which can be very useful for researchers worldwide who want to test their microwave algorithms and prototypes. An example of such an effort is presented in this issue by Rydholm et al. [9], who argue that the high plastic content of 3D-printed materials can introduce additional challenges for microwave tomography reconstructions. Challenges in microwave tomogaphy are also the topic of [10], which discusses the impact of measurement errors and how it can be minimised through a data selection technique prior to inversion.

Microwave tomography is also studied in [11] as a means to monitor thermal ablation, with promising experimental results suggesting that this shows promise as a new medical application where microwave technology can play an important role. A potential therapy application based on microwave technology is also considered in [12], which presents a microwave-based snare inserted into an endoscope, with promising results in simulations and heating experiments of the prototype device. Another interesting application of microwaves in medical diagnostics is presented in [13], which proposes radar technology as means of detecting stress and assessing well-being in a trial with thirty-five healthy volunteers.

One of the most popular medical applications investigated by microwave researchers is breast cancer detection, and this special issue includes three papers on this topic. First, Oliveira et al. present an interesting overview of applying machine learning algorithms as a way to distinguish between benign and malignant tumours, using ultra-wideband (UWB) radar data [14]. UWB imaging is also presented in [15], which succeeds in comparing breast images obtained from patients using UWB radar with X-ray mammography. Promising experimental results are also obtained by the UWB imaging system presented in [16], which assesses the ability of the current prototype to detect tumor-like targets in anatomically complex breast phantoms.

The special issue also includes two review papers, which discuss recent advances and remaining challenges in two important areas of medical-related microwave research. In particular, La Gioia et al. provide a very detailed review of dielectric measurement approaches and results for biological tissues using the open-ended coaxial probe technique [17]. Finally, Yilmaz et al. [18] review recent efforts in the microwave research community to tackle the “holy grail” challenge in diabetes research; that is, non-invasive glucose monitoring.

In summary, this ensemble of articles constitutes a diverse account of current trends and challenges in microwave medical applications. We hope that the readers will find these articles informative and useful, whether their interest lies in algorithms, measurements, or in specific clinical applications. We are also very pleased to see almost all of these papers presenting experimental or clinical results, which suggests that the proposed techniques and applications are being actively pursued through working prototypes. Finally, we are thankful to all the authors for their high-quality contributions, which made the editing of this special issue a thoroughly enjoyable and interesting task.
